# Association of inflammation and nutrition status with all-cause and cardiovascular mortality in individuals with osteoarthritis: NHANES, 1999–2018

**DOI:** 10.3389/fnut.2024.1464414

**Published:** 2024-11-21

**Authors:** Qifang Guo, Yijia Shao, Fei Wang, Wei Zhou, Xinwang Duan

**Affiliations:** Department of Rheumatology and Immunology, The Second Affiliated Hospital, Jiangxi Medical College, Nanchang University, Nanchang, Jiangxi, China

**Keywords:** systemic immune inflammation index (SII), advanced lung cancer inflammation index (ALI), mortality, osteoarthritis, National Health and Nutrition Examination Survey (NHANES), biomarker

## Abstract

**Background:**

Osteoarthritis (OA) is the most prevalent form of arthritis worldwide. Inflammation and nutrition status play crucial roles in the development and progression of OA. The advanced lung cancer inflammation index (ALI) serves as a composite indicator for evaluating inflammation and nutritional status, while the systemic immune inflammation index (SII) is a novel marker for assessing immune-related inflammation. The study aimed to investigate the associations of the ALI and SII with all-cause and cardiovascular mortality among US adults with OA.

**Methods:**

A total of 2,602 individuals aged 20 years and above with OA were included in the study from the National Health and Nutrition Examination Survey (NHANES) spanning from 1999 to 2018. Participants were categorized into higher or lower ALI and SII groups using cut-off values determined by the maximally selected rank statistics method. The Kaplan-Meier analysis, Cox proportional hazards models, and Fine Gray competing risk regression models were employed to assess the associations between the ALI/SII and mortality in OA patients. Additionally, stratified and subgroup analyses were conducted to enhance the robustness of the findings. Furthermore, time-dependent receiver operating characteristic (ROC) analysis was used to evaluate the predictive capacity of ALI and SII for mortality.

**Results:**

Higher SII levels were associated with a 2-fold increase in the risk of all-cause mortality (HR: 2.00, 95% CI: 1.59–2.52, *p* < 0.001), whereas individuals with higher ALI in the OA group exhibited a significantly reduced risk of all-cause mortality (HR: 0.49, 95% CI: 0.39–0.60, *p* < 0.001). Notably, in Model 3, individuals with higher ALI demonstrated a substantially lower risk of cardiovascular mortality (HR: 0.60, 95% CI: 0.44–0.82, *p* < 0.001). Conversely, in fully adjusted models, those with higher SII experienced a significantly higher risk (HR: 1.83, 95% CI: 1.29–2.60, *p* < 0.001). The RCS analysis revealed a J-shaped non-linear relationship between SII levels and all-cause mortality (*p* overall < 0.001; *p* non-linear < 0.001), and an L-shaped non-linear association between ALI levels and all-cause mortality (*p* overall < 0.001; *p* non-linear = 0.002). The time-dependent ROC curves illustrated that ALI and SII displayed a reasonably good and consistent predictive performance for both short- and long-term mortality in OA patients.

**Conclusions:**

Lower ALI and higher SII values were correlated with increased risks of all-cause and cardiovascular mortality among US adults with OA.

## Introduction

Osteoarthritis (OA) is an age-related joint condition impacting articular cartilage and the overall joint structure, causing joint pain, swelling, stiffness, joint deformity, and significant functional limitations, often leading to high disability. The main pathological features included cartilage deterioration, bone resorption, and osteophyte formation (1). OA is becoming increasingly prevalent globally, with substantial impacts on various health outcomes in the US and globally (2). According to data from the National Health Interview Survey, approximately 14 million Americans suffer from symptomatic knee osteoarthritis (KOA) (3). As one of the most common chronic illnesses, OA significantly affects both the physical and mental wellbeing of individuals, while also imposing a substantial financial and medical burden (4). Individuals diagnosed with OA experience a heightened risk of both all-cause and cardiovascular mortality compared to the general population (5, 6). The importance of the management of OA as part of the care of patients with multiple chronic diseases cannot be overlooked, given its impact on disability and functional limitations, as well as high all-cause and cardiovascular mortality rates (7). Therefore, this study intends to provide strategies for the long-term management of patients with arthritis by investigating the correlation between simple and easily accessible biomarkers and mortality in OA and to provide a key reference for the development of targeted interventions in the future.

Low-grade chronic inflammation is believed to be essential in the development of OA, despite its exact pathophysiology remaining unclear (8). In OA, the innate immune system plays a critical role in sustaining persistent, low-grade inflammation (8). Local joint inflammation can directly harm the joint, aggravate the severity of joint symptoms, and increase the loss of cartilage (9). Furthermore, systemic inflammation stimulates local joint inflammation, ultimately resulting in OA (10). Previous research has demonstrated that a close relationship between nutritional status indicators like albumin and body mass index (BMI) and the prognosis of OA patients (11–13). A meta-analysis found that obesity not only increases the risk of osteoarthritis (OA) but also contributes to disease progression and complications, including mortality, particularly when related to cardiovascular comorbidities. The high BMI is a leading factor in OA-related mortality and overall disease burden (14). Albumin level is a useful indicator of nutritional status in clinical populations (15). Low serum albumin was associated with increased mortality and several additional major perioperative complications after total knee arthroplasty for OA (16). Metabolic syndrome and obesity are well-known risk factors for osteoarthritis likely due to increased joint load and the promotion of chronic systemic inflammation (8). Hence, both inflammation and nutritional status impact the prognosis of OA patients. Consequently, the use of a single indicator to assess inflammation or nutritional status for prognosis evaluation may not be comprehensive. There is a critical need for novel predictive indicators that simultaneously consider inflammation and nutritional status to provide a thorough prognosis evaluation for OA patients. This study aims to identify new predictive markers that concurrently evaluate inflammation and nutritional status to comprehensively assess the outcome of OA patients.

The advanced lung cancer inflammation index (ALI) is an innovative composite measure that combines indicators of inflammation and nutritional status by considering parameters such as albumin, BMI, and the neutrophil-to-lymphocyte ratio (NLR). Initially developed to assess the outcome of lung cancer patients, ALI has been expanded for use in various other malignancies like esophageal, colorectal, pancreatic, and gastric cancers (17–22). Due to its capability to comprehensively evaluate both inflammation and nutritional status, ALI's application can be extended to chronic inflammatory conditions such as hypertension, diabetes, heart failure, stroke, and even rheumatoid arthritis (23–27). The systemic immune-inflammation index (SII) is a novel composite marker of systemic inflammation that incorporates neutrophil, lymphocyte, and platelet counts to reflect both pro-inflammatory and anti-inflammatory responses. Initially, it was designed to project longevity and the return of cancer (28, 29). Nonetheless, it is currently uncertain how the ALI and SII relate to the risk of mortality in OA patients.

The study represented a pioneering investigation that explores the associations between the innovative integrated inflammatory and nutritional status markers ALI and SII, and the mortality rates among patients with OA.

## Methods

### Study population

**Figure 1:**
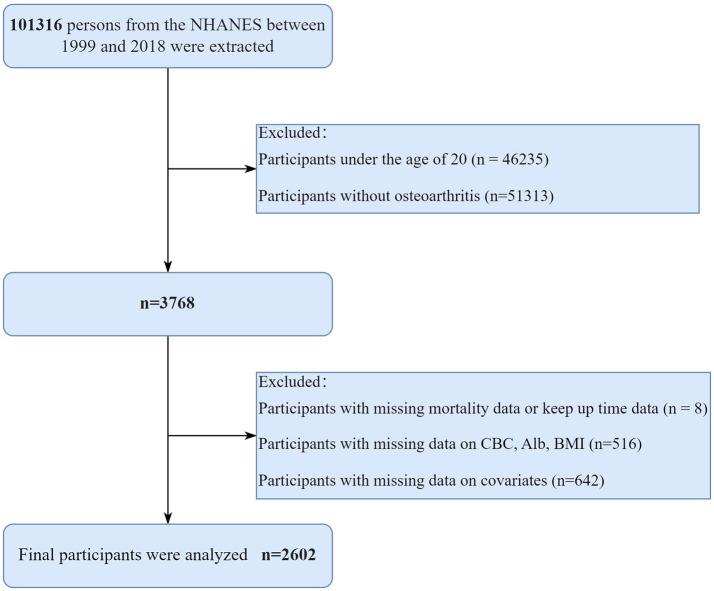
The detailed screening procedure for this study. NHANES, National Health and Nutrition Examination Survey; CBC, complete blood count; Alb, albumin; BMI, Body mass index.

The study utilized public data from the National Health and Nutrition Examination Survey (NHANES) 1999–2018. The NHANES study was approved by the National Center for Health Statistics (NCHS) ethics review board, and all participants gave written informed permission. The study followed the principles of the 1975 Declaration of Helsinki. The study's inclusion criteria were as follows: (1) Participants diagnosed with osteoarthritis; (2) age over 20 years. Some participants were excluded for the following reasons: (1) The data on the level of survival and keep up time are inadequate; (2) Lack of information regarding albumin, BMI, lymphocytes, and neutrophils; (3) Missing details on other covariates. The diagnosis of OA was based on self-reports from participants. Figure 1 displays the detailed screening procedure for this study.

### Measurement of ALI and SII

**Figure 2:**
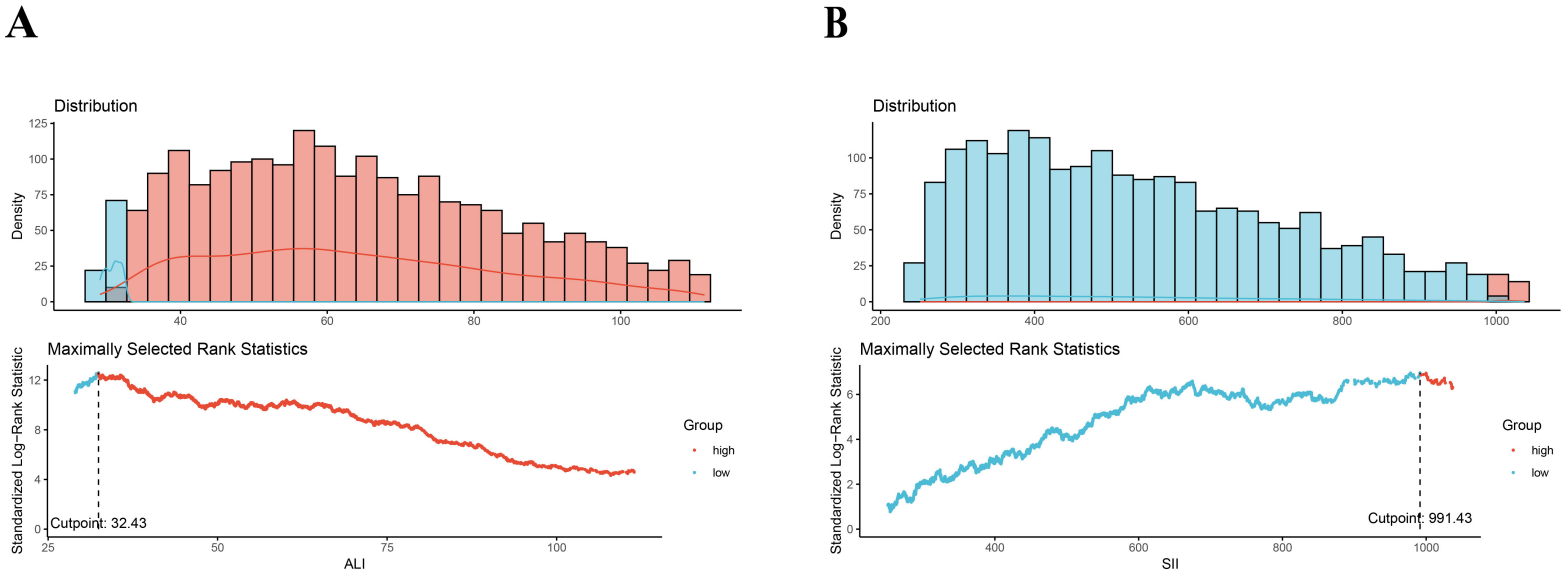
Maximally selected rank statistics method for the ideal cutoff points for the ALI **(A)** and SII **(B)**. SII, systemic immune-inflammation index; SIRI, systemic inflammation response index.

The ALI was calculated using BMI, albumin level (g/dL), and NLR with the following equation: BMI (kg/m^2^) ^*^ albumin/NLR. The NLR was determined by dividing the neutrophil count by the lymphocyte count (30). The SII was calculated by multiplying the platelet count by the neutrophil count and then dividing by the lymphocyte count (28, 29). These values were extracted from peripheral complete blood count (CBC). Thresholds of 32.43 for ALI and 991.43 for SII were determined using a data-driven approach by the maximally selected rank statistics method (MSRSM) (Figure 2). Participants were categorized into two groups based on these cutoff points.

### Definition of mortality

Participant mortality status in the NHANES database was ascertained by accessing the NHANES public-use linked mortality files (LMFs) via a unique personal-level serial number (SEQN) identifier. The most recent update was on December 31, 2019. Specifically, cardiovascular mortality was identified through ICD-10 codes ranging from I00 to I078, and cancer-related mortality was verified for deaths resulting from malignant neoplasms (C00–C97).

### Definition of covariates

The covariates in this study considered demographic characteristics, socioeconomic status, and health conditions. Demographic features comprised age, gender, and race. Socioeconomic status included educational background and poverty-to-income ratio (PIR). Health status included smoking and alcohol consumption status as well as the presence of hypertension or diabetes. Moreover, laboratory data included measurements of total cholesterol and high-density lipoprotein levels.

### Statistical analysis

Due to the complexity of data collection in the NAHNES database, sampling weights were incorporated to ensure the data representativeness, following NHANES recommendations. The specific practice involved combining the MEC examination weights of each cycle (WTMEC4YR, WTMEC2YR, and WTMEPRP). Data analysis was carried using R software version 4.3.2 (R Project for Statistical Computing, Vienna, Austria). Statistical significance level for the tests was set at *p* < 0.05 (two-tailed). For the description of baseline features, persistent variables were presented as mean ± standard deviation, and group differences were compared using analysis of variance (ANOVA). Categorical variables were depicted as frequencies and percentages. Wilcoxon rank-sum test was employed for ordinal categorical variables, while chi-square test was utilized for nominal categorical variables.

Kaplan–Meier (KM) method and univariate Fine Gray competing-risk regression models were conducted to assess the cumulative incidence of all-cause mortality and cardiovascular mortality rate among the higher and lower ALI and SII groups, accounting for the competing risk of non-cardiovascular mortality (27). Hazard ratios (HRs) and 95% confidence intervals (CIs) for mortality were estimated using Cox proportional hazards models and multivariate Fine-Gray models. Three models were employed in this study: Model 1 was without adjustments. Model 2 was modified for age, gender, race, educational background, marital status, and poverty-income ratio. Model 3 was further modified for total cholesterol, high-density lipoprotein, smoking status, drinking status, history of cancer/malignancy, and coronary heart disease. ALI and SII were natural log-transformed for normalization... Restricted cubic spline (RCS) analysis was performed to explore non-linear associations between ALI, SII and mortality in OA patients.

To enhance the robustness of the study, sensitivity and subgroup analyses were conducted. Finally, ROC analyses were employed to evaluate the accuracy of ALI/SII in forecasting short- and long-term survival outcomes at different time points (3-, 5-, 10-, and 15-years).

## Results

### Baseline characteristics

Initially, a total of 101,316 individuals from the NHANES between 1999 and 2018 were included in the analysis. According to the standards for inclusion and exclusion, a total of 2,602 patients with OA from the United States were enrolled in the final study. The detailed flow chart is presented in Figure 1. Among the 2,602 individuals, the average age was 60.26 years, with 63.65% being women. The average ALI was 66.25, the average SII was 620.04, and the average BMI was 30.08 kg/m^2^. Approximately half of the participants (53.88%) were current or former smokers, while more than half (86.99%) were current or former alcohol drinkers. A total of 351 patients (13.49%) had a lower ALI, whereas 294 patients (11.30%) exhibited a higher SII. In comparison to participants with a higher ALI, those with a lower ALI were older, more women, more smokers, had a lower poverty-income ratio, had a lower BMI, and more patients had cancer and cardiovascular disease (CVD) (all *p* < 0.05). However, there were no major distinctions in age, gender, PIR, BMI, smoking status, drinking status and the incidence of cancer and CVD (all *p* > 0.05). Details are provided in Table 1.

**Table 1:** Baseline characteristics of osteoarthritis patients according to the ALI and SII (weighted).

**Characteristic**	**Overall**	**ALI**			**SII**		
	**(*****N** =* **2,602)**	**Higher ALI (*****N** =* **2,251)**	**Lower ALI (*****N** =* **351)**	***P*** **value**	**Higher SII (*****N** =* **294)**	**Lower SII (*****N** =* **2,308)**	***P*** **value**
No. (weighed)	10,311,322	8,991,079	1,320,243		1,192,433	9,118,889	
ALI	66.25 ± 41.53	72.42 ± 40.92	24.21 ± 6.26	< 0.001	30.40 ± 13.90	70.94 ± 41.65	< 0.001
SII	620.04 ± 444.22	522.84 ± 242.63	1,282.01 ± 798.58	< 0.001	1,490.25 ± 732.44	506.25± 202.51	< 0.001
Age (years)	60.26 ± 13.76	59.47 ± 13.47	65.65 ± 14.45	< 0.001	60.91 ± 15.67	60.17 ± 13.49	0.3831
**Age group (%)**				< 0.001			0.0088
20–65 years	1,035 (39.76)	1,416 (62.91)	148 (42.06)		137 (46.71)	897 (38.85)	
≥65 years	1,567 (60.24)	835 (37.09)	203 (57.94)		157 (53.29)	1,411 (61.15)	
**Gender (%)**				0.0234			0.5315
Male	946 (36.35)	800 (35.54)	147 (41.93)		112 (37.99)	834 (36.14)	
Female	1,656 (63.65)	1,451 (64.46)	204 (58.07)		182 (62.01)	1,474 (63.86)	
**Race (%)**				0.0023			0.2270
Mexican American	95 (3.65)	87 (3.86)	8 (2.22)		11 (3.71)	84 (3.64)	
Other Hispanic	73 (2.82)	67 (2.97)	6 (1.80)		9 (3.06)	64 (2.79)	
Non-Hispanic White	2,112 (81.15)	1,800 (79.96)	313 (89.23)		250 (84.93)	1,862 (80.65)	
Non-Hispanic Black	204 (7.86)	190 (8.44)	14 (3.90)		14 (4.71)	191 (8.27)	
Other/multiracial	118 (4.53)	107 (4.77)	10 (2.85)		10 (3.59)	107 (4.65)	
**Educational background (%)**				0.1875			0.0184
< 9th grade	158 (6.08)	134 (5.97)	24 (6.86)		16 (5.6)	142 (6.15)	
9–11th grade	312 (11.98)	259 (11.53)	52 (15.04)		44 (15.09)	267 (11.57)	
High school graduate	676 (25.99)	597 (26.54)	78 (22.23)		70 (23.77)	606 (26.28)	
Some college	826 (31.74)	709 (31.51)	116 (33.28)		110 (37.3)	716 (31.01)	
College graduate or above	630 (24.21)	550 (24.45)	79 (22.58)		54 (18.24)	577 (24.99)	
**Marital status (%)**				< 0.001			0.0513
Married/Living with Partner	1,670 (64.18)	1,243 (55.23)	230 (65.49)		174 (59.11)	1,497 (64.84)	
Widowed/Divorced/ Separated/Never Married	932 (35.82)	1,007 (44.77)	121 (34.51)		120 (40.89)	811 (35.16)	
Poverty–income ratio	3.01 ± 1.59	3.05 ± 1.60	2.74 ± 1.54	< 0.001	2.87 ± 1.50	3.03 ± 1.60	0.1030
**Poverty–income ratio group (%)**				< 0.001			0.0004
< 1.29	502 (19.3)	429 (19.07)	73 (20.85)		53(18.12)	449 (19.45)	
1.30–3.49	1,002 (38.5)	836 (37.12)	168 (47.87)		143(48.66)	858 (37.17)	
≥3.50	1,098 (42.21)	986 (43.81)	109 (31.28)		98(33.22)	1,001 (43.38)	
BMI (kg/m^2^)	30.08 ± 7.03	30.67 ± 7.07	26.10 ± 5.26	< 0.001	30.65 ± 7.92	30.01 ± 6.90	0.1365
Albumin (g/dL)	4.19 ± 0.33	4.21 ± 0.32	4.09 ± 0.36	< 0.001	4.07 ± 0.37	4.21 ± 0.32	< 0.001
Neutrophil count (K/uL)	4.37 ± 1.69	4.12 ± 1.44	6.06 ± 2.24	< 0.001	6.76 ± 2.28	4.06 ± 1.31	< 0.001
Lymphocyte count (K/uL)	2.08 ± 1.30	2.19 ± 1.35	1.34 ± 0.46	< 0.001	1.57 ± 0.65	2.14 ± 1.35	< 0.001
Platelet count (K/uL)	258.28 ± 74.96	257.40 ± 69.24	264.33 ± 105.80	0.1151	327.41 ± 108.01	249.25 ± 64.20	< 0.001
NLR	2.39 ± 1.42	2.19 ± 1.35	4.84 ± 2.28	< 0.001	4.81 ± 2.41	2.07 ± 0.81	< 0.001
Total cholesterol (mmol/L)	5.24 ± 1.11	5.28 ± 1.11	4.95 ± 1.07	< 0.001	5.09 ± 1.09	5.26 ± 1.11	0.0131
High density lipoprotein (mmol/L)	1.41 ± 0.42	1.41 ± 0.41	1.46 ± 0.44	0.0417	1.41 ± 0.42	1.41 ± 0.42	0.8161
**Smoking status (%)**				0.0448			0.2966
Current	455 (17.49)	388 (17.23)	67 (19.25)		52 (17.53)	404 (17.49)	
Former	947 (36.39)	804 (35.72)	144 (40.97)		118 (40.19)	828 (35.89)	
Never	1,200 (46.12)	1,059 (47.05)	140 (39.78)		124 (42.29)	1,076 (46.62)	
**Drinking status (%)**				0.9777			0.4210
Current	1,806 (69.43)	1,564 (69.5)	242 (68.94)		208 (70.62)	1,599 (69.27)	
Former	457 (17.56)	395 (17.53)	62 (17.76)		55 (18.69)	402 (17.41)	
Never	339 (13.02)	292 (12.98)	47 (13.3)		31 (10.69)	307 (13.32)	
**Cancer/malignancy**				0.0101			0.3395
Yes	470 (18.07)	390 (17.33)	81 (23.13)		47 (16.08)	423 (18.33)	
No	2,132 (81.93)	1,861 (82.67)	270 (76.87)		247 (83.92)	1,885 (81.67)	
**Coronary heart disease (%)**				0.0241			0.9304
Yes	215 (8.25)	175 (7.78)	40 (11.43)		25 (8.38)	190 (8.23)	
No	2,387 (91.75)	2,076 (92.22)	311 (88.57)		269 (91.62)	2,118 (91.77)	

ALI, advanced lung cancer inflammation index; SII, Systemic immune inflammation index.

### Associations of the ALI and SI with mortality

**Figure 3:**
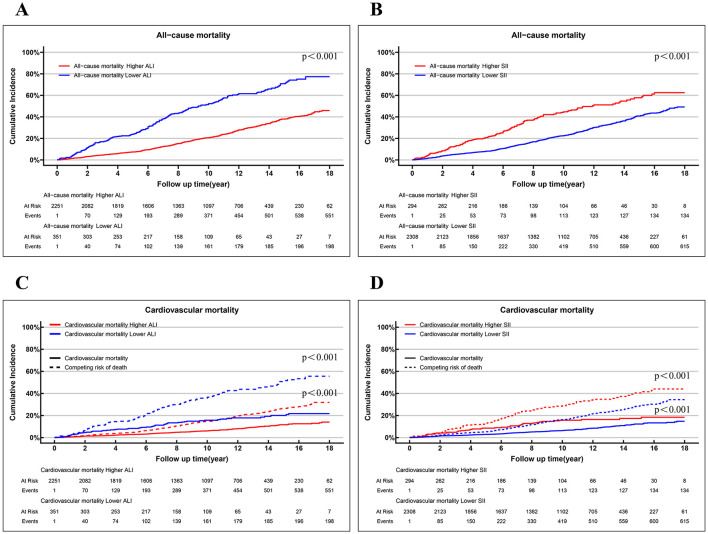
Cumulative incidence curves demonstrating how the ALI/SII affects mortality results. Kaplan-Meier curves for all-cause mortality, stratified by ALI **(A)** and SII **(B)**. Competing risk analysis for cardiovascular mortality and competing risk of death for the ALI **(C)** and SII **(D)** groups. OA, osteoarthritis; ALI, Advanced Lung Cancer Inflammation Index; SII, systemic immune–inflammation index.

Among the 2,602 patients with osteoarthritis, 636 all-cause deaths (28.82% of the population) and 226 cardiovascular-related deaths (8.89% of the sample) were recorded over an average follow-up period of 9.42 years (IQR 4.94–12.58 years). The KM curves and univariate Fine–Gray models based on ALI and SII values among adults with OA are presented in Figure 3. Individuals with lower ALI and higher SII levels demonstrated significantly higher all-cause mortality compared to those with higher ALI and lower SII values (both *p* < 0.001). Similarly, lower ALI values and higher SII values were significantly associated with an increased cumulative probability of cardiovascular mortality during the follow-up period, compared to higher ALI and lower SII values (both *p* < 0.001).

Table 2 presents the results of four Cox models that illustrate the associations between inflammation and nutrition status and both all-cause and cardiovascular mortality. Model 3 included a broader range of covariates to better mitigating account for confounding variables, with a focus on the results derived from the model. The subgroup with higher ALI exhibited significantly lower all-cause mortality rates compared to those with low ALI (HR: 0.49, 95% CI: 0.39–0.60, *p* < 0.001). Higher SII levels were associated with a 2-fold increased risk of all-cause mortality (HR: 2.00, 95% CI: 1.59–2.52, *p* < 0.001). In the multivariate Fine-Gray models of model 3, the higher-ALI group had a considerably reduced risk of cardiovascular mortality (HR: 0.60, 95% CI: 0.44–0.82, *p* < 0.001) while the higher-SII group exhibited a considerably higher risk in fully adjusted models (HR: 1.83, 95% CI: 1.29–2.60, *p* < 0.001).

**Table 2:** Hazard ratios of all-cause mortality and cardiovascular mortality according to the ALI and SII among patients with osteoarthritis.

**Characteristic**	**Model 1**		**Model 2**		**Model 3**	
	**HR (95% CI)**	***P*** **value**	**HR (95% CI)**	***P*** **value**	**HR (95% CI)**	***P*** **value**
**All-cause mortality**						
ALI group	0.33 (0.26, 0.41)	< 0.001	0.47 (0.39, 0.58)	< 0.001	0.49 (0.39, 0.60)	< 0.001
SII group	1.99 (1.53, 2.57)	< 0.001	1.91 (1.51, 2.41)	< 0.001	2.00 (1.59, 2.52)	< 0.001
**Cardiovascular mortality**						
ALI group	0.36 (0.27, 0.49)	< 0.001	0.59 (0.43, 0.81)	0.001	0.60 (0.44, 0.82)	< 0.001
SII group	2.09 (1.49, 2.92)	< 0.001	1.74 (1.23, 2.45)	0.001	1.83 (1.29, 2.60)	< 0.001
**Competing risk of death**						
ALI group	0.33 (0.27, 0.40)	< 0.001	0.48 (0.39, 0.59)	< 0.001	0.49 (0.40, 0.60)	< 0.001
SII group	1.95 (1.55, 2.44)	< 0.001	1.73 (1.38, 2.18)	< 0.001	1.75 (1.39, 2.21)	< 0.001

Cox proportional hazards models were employed to evaluate the relationships between ALL and SII with all-cause mortality rate. Multivariate Fine Gray competing risk regression models were employed to evaluate the relationships between ALL and SII with cardiovascular and non-cardiovascular mortality rate. Competing risk of mortality was defined as fatalities caused by factors other than cardiovascular disease. Model 1 was not altered. Model 2 was adjusted for age, gender, race, educational background, marital status, and the poverty-income ratio. Model 3 was adjusted for age, gender, race, educational background, marital status, poverty-income ratio, total cholesterol, high density lipoprotein, smoking status, alcohol consumption, cancer/malignancy, and coronary heart disease. ALI, advanced lung cancer inflammation index; SII, Systemic immune inflammation index; HR, hazard ratios; CI, confidence interval.

### Non-linear relationships

**Figure 4:**
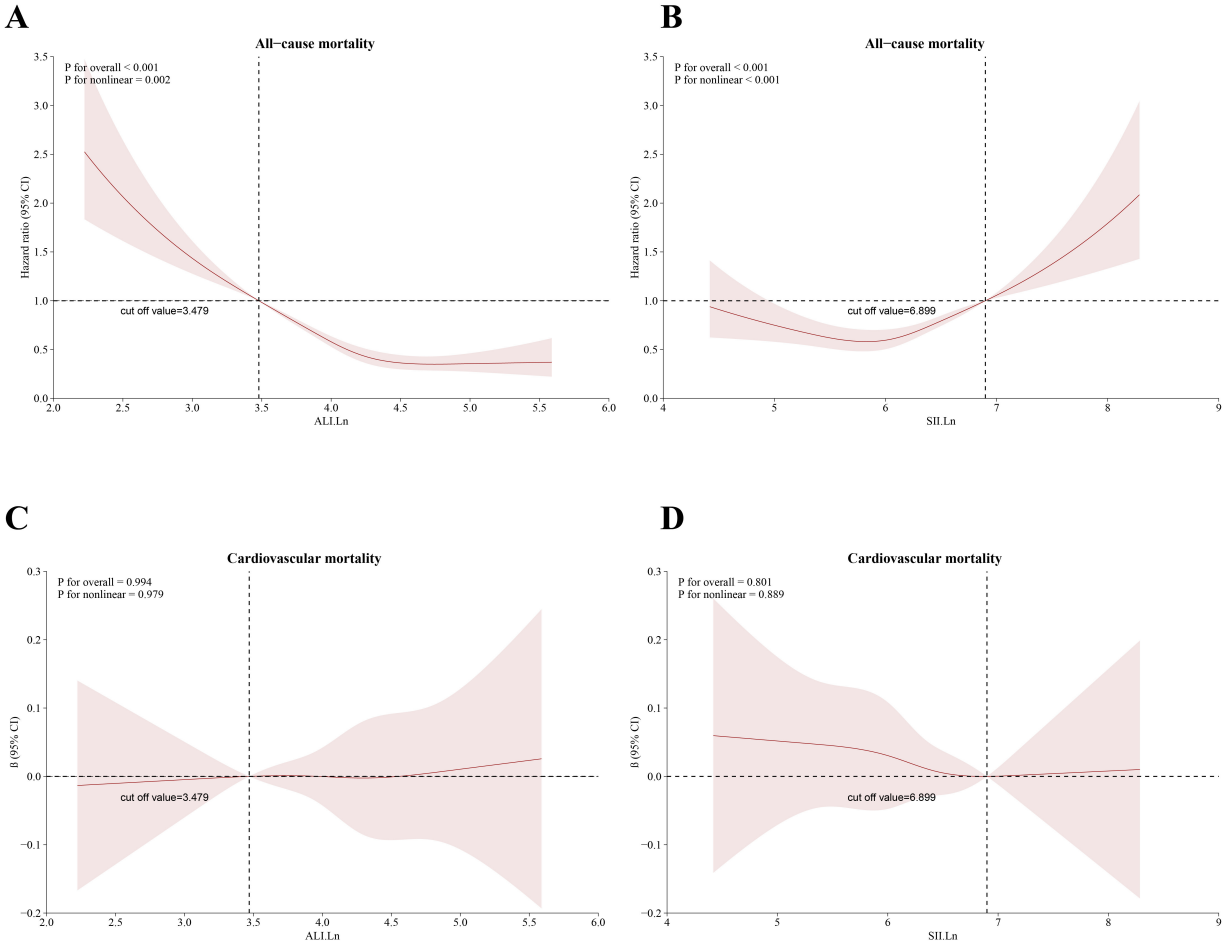
Restricted cubic spline for the relationship between death and the ALI and SII. According to the ALI and SII, **(A, B)** represent all-cause mortality; **(C, D)** represent cardiovascular mortality. The 95% CI is shown by the transparent area, while the HR is indicated by the red line. Model 3 was used to alter these analyses. ALI, Advanced Lung Cancer Inflammation Index; SII, systemic immune–inflammation index.

There existed an L-shaped non-linear correlation among ALI and all-cause mortality (*p* overall < 0.001; *p* non-linear = 0.002) (Figure 4A), while a J-shaped non-linear association was observed between SII levels and mortality (*p* overall < 0.001; *p* non-linear < 0.001) (Figure 4B). Conversely, a linear correlation was found between ALI, the SII, and cardiovascular mortality (*p* overall > 0.05; *p* non-linear > 0.05) (Figures 4C, D).

### Subgroup and sensitivity analyses

Table 3 displays the associations of the ALI and SII with all-cause mortality in subgroup analysis. The results indicated that low ALI and high SII were associated with an increased risk of all-cause mortality in most subgroups. Notably, the relationship between SII and all-cause mortality was insignificant in individuals with coronary heart disease. The relationships between cardiovascular rates and SII were observed across subgroups (Table 4). However, with regards to cardiovascular mortality, significant interactions were only found with age and PIR subgroups, while other subgroups did not show any notable interactions. Furthermore, sensitivity analysis demonstrated no significant changes in results when analyzing individuals who died from cancer (*n* = 2,451) (Supplementary Table S1), deaths due to other/unspecified causes (*n* = 2,414) (Supplementary Table S2), individuals under 40 years of age (*n* = 2,446) (Supplementary Table S3), or those with < 3 years of follow-up (*n* = 2,230) (Supplementary Table S4).

**Table 3:** Subgroup analysis investigation of the relationships between the ALI/SII and all-cause mortality among osteoarthritis patients.

**Subgroup**	**ALI**		**SII**	
	**HR (95% CI)**	***P*** **value**	**HR (95% CI)**	***P*** **value**
**Age group**				
20–65 years	0.36 (0.24, 0.54)	< 0.001	1.66 (1.04, 2.64)	0.0334
≥65 years	0.40 (0.34, 0.48)	< 0.001	2.10 (1.71, 2.58)	< 0.001
**Gender**				
Male	0.36 (0.24, 0.54)	< 0.001	2.46 (1.88, 3.23)	< 0.001
Female	0.36 (0.29, 0.46)	< 0.001	1.70 (1.31, 2.20)	< 0.001
**Marital status**				
Married/Living with Partner	0.34 (0.27, 0.43)	< 0.001	2.15 (1.65, 2.81)	< 0.001
Widowed/Divorced/Separated/Never Married	0.35 (0.28, 0.44)	< 0.001	1.77 (1.36, 2.30)	< 0.001
**Poverty–income ratio group**				
< 2.49	0.36 (0.29, 0.44)	< 0.001	2.46 (1.84, 3.28)	< 0.001
≥2.50	0.32 (0.25, 0.41)	< 0.001	1.69 (1.32, 2.16)	< 0.001
**Smoking status**				
Current	0.37 (0.24, 0.58)	< 0.001	1.74 (1.03, 2.94)	0.0377
Former	0.31 (0.24, 0.39)	< 0.001	2.30 (1.77, 3.00)	< 0.001
Never	0.36 (0.28, 0.47)	< 0.001	1.72 (1.26, 2.35)	< 0.001
**Drinking status**				
Current	0.32 (0.26, 0.39)	< 0.001	2.13 (1.69, 2.68)	< 0.001
Former	0.41 (0.28, 0.58)	< 0.001	1.82 (1.20, 2.77)	0.0047
Never	0.33 (0.22, 0.48)	< 0.001	1.78 (1.09, 2.91)	0.0204
**Cancer/malignancy**				
Yes	0.36 (0.27, 0.49)	< 0.001	2.57 (1.80, 3.68)	< 0.001
No	0.34 (0.28, 0.41)	< 0.001	1.90 (1.52, 2.37)	< 0.001
**Coronary heart disease**				
Yes	0.37 (0.24, 0.57)	< 0.001	1.53 (0.89, 2.64)	0.1219
No	0.33 (0.28, 0.40)	< 0.001	2.11 (1.73, 2.58)	< 0.001

ALI, advanced lung cancer inflammation index; SII, Systemic immune inflammation index; HR, hazard ratios; CI, confidence interval.

**Table 4:** Subgroup analysis of the relationships between the ALI/SII and cardiovascular mortality among osteoarthritis patients.

**Subgroup**	**ALI**		**SII**	
	**HR (95% CI)**	***P*** **value**	**HR (95% CI)**	***P*** **value**
**Age group**				
20–65 years	0.45 (0.21, 0.97)	0.0401	5.3 (2.3, 12.0)	< 0.001
≥65 years	0.84 (0.60, 1.16)	0.2816	1.2 (0.9, 1.8)	0.2400
**Gender**				
Male	0.75 (0.49, 1.15)	0.1874	1.6 (1.0, 2.5)	0.0600
Female	0.77 (0.50, 1.19)	0.2429	1.6 (1.0, 2.6)	0.0560
**Marital status**				
Married/Living with Partner	0.88 (0.54, 1.44)	0.6138	1.8 (1.1, 3.0)	0.0320
Widowed/Divorced/Separated /Never Married	0.72 (0.49, 1.06)	0.0987	1.4 (0.9, 2.1)	0.1490
**Poverty–income ratio group**				
< 2.49	0.61 (0.37, 0.98)	0.0424	2.4 (1.4, 4.0)	0.0010
≥2.50	0.87 (0.59, 1.28)	0.4784	1.2 (0.8, 1.9)	0.3330
**Smoking status (%)**				
Current	0.58 (0.26, 1.31)	0.1892	4.7 (1.9, 11.6)	< 0.001
Former	0.77 (0.51, 1.18)	0.2362	1.1 (0.7, 1.8)	0.7430
Never	0.82 (0.49, 1.37)	0.4529	1.7 (1.0, 3.0)	0.070
**Drinking status (%)**				
Current	0.77 (0.52, 1.13)	0.1829	1.4 (0.9, 2.2)	0.1410
Former	0.74 (0.39, 1.40)	0.3500	2.2 (1.1, 4.5)	0.0290
Never	0.74 (0.37, 1.48)	0.3891	1.8 (0.8, 4.0)	0.1310
**Cancer/malignancy**				
Yes	0.56 (0.32, 0.97)	0.0385	1.7 (0.9, 3.3)	0.0970
No	0.85 (0.59, 1.23)	0.3900	1.5 (1.0, 2.2)	0.0420
**Coronary heart disease (%)**				
Yes	0.57 (0.28, 1.16)	0.1194	4.7 (2.2, 9.8)	< 0.001
No	0.74 (0.53, 1.03)	0.0761	1.4 (1.0, 2.1)	0.0670

ALI, advanced lung cancer inflammation index; SII, Systemic immune inflammation index; HR, hazard ratios; CI, confidence interval.

### Predictive performance

**Figure 5:**
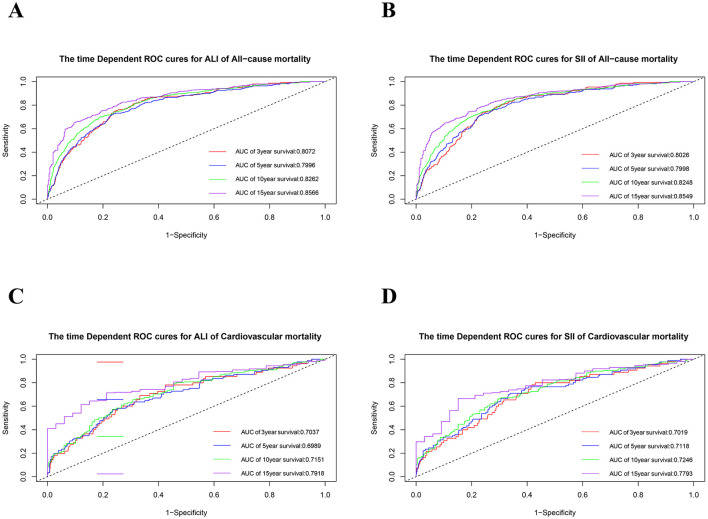
The time-dependent ROC curves for 3-, 5-, 10-, and 15- year survival predictions. **(A)** ALI for all-cause mortality; **(B)** SII for all-cause mortality; **(C)** ALI for cardiovascular mortality; **(D)** SII for cardiovascular mortality. ROC, receiver operating characteristic; SII, systemic immune-inflammation index; SIRI, systemic inflammation response index.

To evaluate the predictive performance of ALI and SII in patients with OA, time-dependent ROC (Figure 5) and AUC curves (Supplementary Figure S1). were generated based on the results of the model 3 Cox regression analysis. The AUCs for the ALI were 0.8072, 0.7996, 0.8262, and 0.8566 at 3-, 5-, 10-, and 15-years of all-cause mortality, respectively. Similarly, the SII showed AUCs values of 0.8026, 0.7998, 0.8248, and 0.8549 for the corresponding time points. For cardiovascular mortality predictions, the ALI had AUCs of 0.7037, 0.6989, 0.7151, and 0.7918 at 3-, 5-, 10-, and 15-years intervals, respectively. Conversely, the SII demonstrated AUC values of 0.7019, 0.7118, 0.7246, and 0.7793 at the same time intervals (Figure 5). The time-dependent ROC and AUC curves demonstrated that both ALI and SII exhibited moderate and consistent performance in predicting mortality.

## Discussion

This is a large sample study aimed at providing easy and accessible monitoring markers for the long-term management of OA and providing evidence-based evidence for policy makers and clinicians. Based on a sizable cohort, this investigation was the first to explore the connections between ALI and SII and cardiovascular as well as all-cause mortality in OA patients. An increase in ALI was associated with reduced all-cause and cardiovascular mortality after multivariate adjustments. On the other hand, there was a strong correlation between an increase SII and increased cardiovascular and all-cause mortality. The data also revealed a J-shaped non-linear association between SII levels and all-cause mortality, as well as an L-shaped non-linear link between ALI levels and all-cause mortality. These results highlight the significance of ALI and SII in assessing the risk and predicting the prognosis of osteoarthritis (OA) patients. Furthermore, ROC analysis confirmed the predictive capability of ALI and SII in forecasting survival outcomes in OA patients. The AUC values for predicting all-cause mortality at 3-, 5-, 10-, and 15-years were 0.8072, 0.7996, 0.8262, and 0.8566 for ALI, and 0.8026, 0.7998, 0.8248, and 0.8549 for SII, respectively.

Patients with OA experience higher all-cause death rates than the general population, especially if they have a history of cardiovascular disease, which is a major risk factor (31). Recent evidence suggests that OA increases the risk of developing CVD (32, 33). While OA was traditionally believed to result only from cartilage deterioration causing joint damage, a more modern perspective emphasizes the role of both systemic and local inflammation in causing cartilage damage (34). OA is now understood as a joint disease involving inflammation of the synovium (synovitis), with inflammatory cell infiltration being a key characteristic (35–37). Additionally, synovitis in OA is associated with more severe joint symptoms, greater cartilage loss, reduced mobility, and higher radiographic grades (38, 39). Obesity and metabolic syndrome are significant modifiable risk factors for both the onset and advancement of OA (40, 41).

As an index integrating inflammation and nutrition, the prognostic value of ALI has been demonstrated in patients with various types of cancer (17, 19, 21). Previous research has established ALI as a reliable marker for predicting outcomes in operable non-small cell lung cancer patients (42). Recent studies have indicated the utility of ALI in assessing prognosis and the protective effects in patients with rheumatoid arthritis (27), which is consistent with our findings. The L-shaped non-linear link between ALI levels and all-cause mortality in OA patients can be explained from several perspectives. Firstly, the NLR plays a crucial role. High levels of neutrophils indicated non-specific inflammation, while low levels of lymphocytes indicated impaired immune function (43). Therefore, a higher NLR may reflect immune activity during chronic inflammation (44). In OA, the inflammation is typically chronic and low-grade, involving primarily innate immune responses with some adaptive involvement (45). The infiltration of immune cells into the synovial membrane leads to synovitis in the early stages of OA. Neutrophils are the first immune cells to post the synovium following joint damage, promoting tissue degradation through neutrophil elastase (NE) action, osteophyte formation, and the production of inflammatory cytokines and chemokines (46). Meanwhile, lymphocytes also accumulate in the synovium with impaired T and B cell function observed in OA patients compared to healthy individuals, indicating a dysfunctional immune response that may contribute significantly to OA pathogenesis (47–49). Secondly, serum albumin serves as a widely used marker for assessing nutritional status. Research has shown higher levels of serum albumin in OA patients (50). Evidence from a Mendelian randomization (MR) study supports that high levels of retinol and albumin may have a preventive impact on the incidence of osteoarthritis (12). Serum albumin has been implicated in pain reduction and effective OA management by promoting anti-inflammatory prostaglandin production, assisting in inflammation resolution, supporting cartilage healing, and aiding in symptom improvement (51). Low albumin levels may predict poorer health outcomes due to its role as a marker for systemic inflammation and nutritional status, which are crucial factors in managing OA and its comorbidities, such as cardiovascular diseases (52). Thirdly, obesity has been linked to unfavorable outcomes in OA patients. Enhanced BMI significantly increases the risk of developing radiographic and clinical hand OA, as indicated in recent meta-analyses (14, 53). Obesity is a major cause of OA, driven by multiple factors beyond biomechanical changes. Additionally, adipose tissue induces the production of inflammatory mediators and cartilage degradation factors, which contribute to cartilage deterioration and the progression of OA (54, 55). A study on elderly patients revealed that obesity not only poses a risk for OA but also for cardiovascular disease (13). Research also suggested that extreme BMIs—both low and high—are linked to worse outcomes, including higher mortality (56).

The SII has been seen associated with higher mortality rates in individuals with various diseases (57, 58). Osteoarthritis is becoming regarded as a low-grade inflammatory condition that can be impacted by platelets. Elevated platelet levels, which frequently reflect systemic inflammation, are associated with OA severity and may imply a higher risk of mortality, particularly from cardiovascular problems, which are common in OA patients (59). There is a growing understanding of the significant role platelets play in inflammatory and immune responses. Platelet activation results in increasing immune responses, contributing to the degradation of cartilage and bone in OA (60). Platelet counts outside the normal range (either low or high) predicted increased all-cause and cardiovascular mortality in the general population and the elderly, which may explain the J-shaped association between SII and all-cause mortality of OA (61). The SII integrates three distinct types of inflammatory cells, demonstrating their interconnected impacts and cumulative influence. The approach avoids the limitations of single blood cell measures, which can be influenced by factors affecting cell volume, thereby providing a more comprehensive insight into the overall inflammatory condition. Based on the results, ALI and SII are expected to be predictors for risk stratification in patients with osteoarthritis. Our results showed that elevated ALI levels (>32.23) and lower SII values (< 991.43) are associated with consistently lower mortality rates among OA patients. Therefore, more attention and management should be strengthened for this population. The study revealed that the relationships between ALI, SII and mortality outcomes in individuals with OA varied by subgroup and were not totally consistent. In terms of all-cause events, the association with lower ALI levels and higher SII values was most pronounced in individuals aged 20–65 years, males, former smokers, and current drinkers. This could be explained by the higher prevalence of OA in females compared to and in older individuals compared to younger ones (62). Notably, the inflammatory and nutritional status of patients with osteoarthritis changes over time, and these changes may lead to anti-inflammatory and pro-inflammatory imbalances, comorbidities, and increased resistance to medications (63). Therefore, dynamic monitoring of ALI and SII and assessing their impact on OA mortality may offer deeper insights.

This research has several important implications. Firstly, an important advantage of this research is the use of a substantial, nationwide cohort of US people that was tracked over time. Secondly, we enhanced the robustness of our results by conducting extensive subgroup and sensitivity analyses. Particularly, our findings highlight the prospective value of the ALI and SII as predictive factors for assessing the likelihood of death in OA patients. These variables, which can be quite easily obtained from CBC, liver function tests, and basic body measurements without additional expense, could assist healthcare professionals in identifying OA patients at a heightened risk of mortality, enabling targeted interventions to reduce risks. In order to lessen confounding effects, we included total cholesterol and HDL as factors since hyperlipidemia is a risk factor for OA.

Nonetheless, there are several limitations. Firstly, the impact of dynamic changes in ALI and SII on the mortality rate of OA were not evaluated in this study. Secondly, while we adjusted for the majority of possible variables, residual confounding due to unmeasured factors may still be present. Genetic predisposition, psychological stress, and occupational factors can all influence inflammatory nutritional status, but could not be adjusted in our study. The effects of these confounding factors should be attended to in future database development and clinical studies to improve the accuracy of the model estimates. Thirdly, potential bias could have arisen due to the reliance on self-reported OA diagnoses by patients. The concordance rate between self-reported OA and clinically well-defined OA was 81%, so the OA diagnosis can be confirmed by combining clinical assessment and imaging data, such as bone density and digital radiography. Fourthly, the data extracted from the NHANES database are mainly from household interviews and questionnaires, then there may be a selection bias by excluding individuals with severe disability who may not participate in NHANES. Therefore, it is imperative to validate our discoveries in varied cohorts and explore the fundamental mechanisms further.

## Conclusion

In conclusion, our research showed that lower ALI levels and higher SII values, reflecting increased systemic inflammation and poor nutritional status, are independently associated with higher risks of all-cause and cardiovascular mortality in US adults with osteoarthritis. There was a J-shaped non-linear link between SII values and all-cause mortality and an L-shaped non-linear relationship between ALI levels and all-cause mortality in OA patients. However, to better understand the complex interactions between inflammatory and nutritional biomarkers and survival outcomes in people with OA, more research is necessary.

## Data Availability

The datasets presented in this study can be found in online repositories. The names of the repository/repositories and accession number(s) can be found at: https://www.cdc.gov/nchs/data-linkage/mortality.htm.
