# Supplementary material for: Association of inflammation and nutrition status with all-cause and cardiovascular mortality in individuals with osteoarthritis: NHANES, 1999–2018
Source: Front Nutr. 2024 Nov 21;11:1464414. doi: 10.3389/fnut.2024.1464414 (PMC11617147; doi:10.3389/fnut.2024.1464414)
Supplement: Supplementary file 1 [file Data_Sheet_1.docx]

**Supplementary Material**

**Table S1 HR (95% CIs) for mortality after excluding any participants who died from cancer (n = 2451).**

| **Characteristic** | **Model 1** | |  | **Model 2** | |  | **Model 3** | |
| --- | --- | --- | --- | --- | --- | --- | --- | --- |
|  | **HR (95% CI)** | **P value** |  | **HR (95% CI)** | **P value** |  | **HR (95% CI)** | **P value** |
| **All-cause mortality** |  |  |  |  |  |  |  |  |
| ALI group | 0.30（0.24,0.39）） | <0.001 |  | 0.45（0.36,0.57）） | <0.001 |  | 0.46（0.36,0.57）） | <0.001 |
| SII group | 2.12（1.60,2.80）） | <0.001 |  | 2.06（1.60,2.66）） | <0.001 |  | 2.18（1.70,2.81）） | <0.001 |
| **Cardiovascular mortality** |  |  |  |  |  |  |  |  |
| ALI group | 0.36 (0.26, 0.48) | <0.001 |  | 0.61 (0.44, 0.83) | 0.002 |  | 0.61 (0.44, 0.84) | 0.002 |
| SII group | 2.07 (1.48, 2.89) | <0.001 |  | 1.72 (1.22, 2.42) | 0.002 |  | 1.82 (1.28, 2.58) | <0.001 |
| **Competing risk of death** |  |  |  |  |  |  |  |  |
| ALI group | 0.27 (0.21, 0.33) | <0.001 |  | 0.42 (0.33, 0.53) | <0.001 |  | 0.42 (0.33, 0.53) | <0.001 |
| SII group | 2.24 (1.73, 2.89) | <0.001 |  | 1.72 (1.22, 2.42) | <0.001 |  | 1.98 (1.52, 2.58) | <0.001 |

Cox proportional hazards models were employed to evaluate the relationships between ALL and SII with all-cause mortality rate. Multivariate Fine Gray competing risk regression models were employed to evaluate the relationships between ALL and SII with cardiovascular and non-cardiovascular mortality rate. Competing risk of mortality was defined as fatalities caused by factors other than cardiovascular disease. Model 1 was not altered. Model 2 was adjusted for age, gender, race, educational background, marital status, and the poverty-income ratio. Model 3 was adjusted for age, gender, race, educational background, marital status, poverty-income ratio, total cholesterol, high density lipoprotein, smoking status, alcohol consumption, cancer/malignancy, and coronary heart disease.

Abbreviations: ALI, advanced lung cancer inflammation index; SII, Systemic immune inflammation index; HR, hazard ratios; CI, confidence interval.

**Table S2 HR (95% CIs) for mortality after excluding participants with other/unspecified causes of death (n = 2414)**

| **Characteristic** | **Model 1** | |  | **Model 2** | |  | **Model 3** | |
| --- | --- | --- | --- | --- | --- | --- | --- | --- |
|  | **HR (95% CI)** | **P value** |  | **HR (95% CI)** | **P value** |  | **HR (95% CI)** | **P value** |
| **All-cause mortality** |  |  |  |  |  |  |  |  |
| ALI group | 0.31（0.24,0.40）） | <0.001 |  | 0.43（0.34,0.54） | <0.001 |  | 0.44（0.35,0.56）） | <0.001 |
| SII group | 2.18（1.63,2.91）） | <0.001 |  | 2.31（1.78,3.01）） | <0.001 |  | 2.42（1.86,3.15）） | <0.001 |
| **Cardiovascular mortality** |  |  |  |  |  |  |  |  |
| ALI group | 0.34 (0.25, 0.46) | <0.001 |  | 0.50 (0.36, 0.68) | <0.001 |  | 0.50 (0.36, 0.69) | <0.001 |
| SII group | 2.18 (1.56, 3.05) | <0.001 |  | 2.19 (1.56, 3.09) | <0.001 |  | 2.25 (1.59, 3.20) | <0.001 |
| **Competing risk of death** |  |  |  |  |  |  |  |  |
| ALI group | 0.31 (0.24, 0.39) | <0.001 |  | 0.42 (0.33, 0.54) | <0.001 |  | 0.43 (0.33, 0.55) | <0.001 |
| SII group | 2.06 (1.55, 2.73) | <0.001 |  | 2.12 (1.59, 2.82) | <0.001 |  | 2.15 (1.61, 2.87) | <0.001 |

Cox proportional hazards models were employed to evaluate the relationships between ALL and SII with all-cause mortality rate. Multivariate Fine Gray competing risk regression models were employed to evaluate the relationships between ALL and SII with cardiovascular and non-cardiovascular mortality rate. Competing risk of mortality was defined as fatalities caused by factors other than cardiovascular disease. Model 1 was not altered. Model 2 was adjusted for age, gender, race, educational background, marital status, and the poverty-income ratio. Model 3 was adjusted for age, gender, race, educational background, marital status, poverty-income ratio, total cholesterol, high density lipoprotein, smoking status, alcohol consumption, cancer/malignancy, and coronary heart disease.

Abbreviations: ALI, advanced lung cancer inflammation index; SII, Systemic immune inflammation index; HR, hazard ratios; CI, confidence interval.

## Table S3 HR (95% CIs) for mortality after excluding participants younger than 40 years (n = 2446)

| **Characteristic** | **Model 1** | |  | **Model 2** | |  | **Model 3** | |
| --- | --- | --- | --- | --- | --- | --- | --- | --- |
|  | **HR (95% CI)** | **P value** |  | **HR (95% CI)** | **P value** |  | **HR (95% CI)** | **P value** |
| **All-cause mortality** |  |  |  |  |  |  |  |  |
| ALI group | 0.32（0.25,0.40）） | <0.001 |  | 0.48（0.39,0.59） | <0.001 |  | 0.49（0.40,0.61）） | <0.001 |
| SII group | 2.24（1.73,2.93） | <0.001 |  | 1.93（1.53,2.43） | <0.001 |  | 2.00（1.59,2.52） | <0.001 |
| **Cardiovascular mortality** |  |  |  |  |  |  |  |  |
| ALI group | 0.36 (0.26, 0.48) | <0.001 |  | 0.61 (0.45, 0.83) | 0.0020 |  | 0.62 (0.45, 0.85) | 0.0033 |
| SII group | 2.25 (1.60, 3.16) | <0.001 |  | 1.69 (1.19, 2.39) | 0.0032 |  | 1.78 (1.25, 2.53) | 0.0014 |
| **Competing risk of death** |  |  |  |  |  |  |  |  |
| ALI group | 0.32 (0.26, 0.39) | <0.001 |  | 0.48 (0.40, 0.59) | <0.001 |  | 0.49 (0.40, 0.61) | 0.0033 |
| SII group | 2.15 (1.71, 2.70) | <0.001 |  | 1.73 (1.37, 2.18) | <0.001 |  | 1.75 (1.38, 2.21) | <0.001 |

Cox proportional hazards models were employed to evaluate the relationships between ALL and SII with all-cause mortality rate. Multivariate Fine Gray competing risk regression models were employed to evaluate the relationships between ALL and SII with cardiovascular and non-cardiovascular mortality rate. Competing risk of mortality was defined as fatalities caused by factors other than cardiovascular disease. Model 1 was not altered. Model 2 was adjusted for age, gender, race, educational background, marital status, and the poverty-income ratio. Model 3 was adjusted for age, gender, race, educational background, marital status, poverty-income ratio, total cholesterol, high density lipoprotein, smoking status, alcohol consumption, cancer/malignancy, and coronary heart disease.

Abbreviations: ALI, advanced lung cancer inflammation index; SII, Systemic immune inflammation index; HR, hazard ratios; CI, confidence interval.

**Table S4 HR (95% CIs) for mortality after excluding participants with <3 years of follow-up (n = 2230)**

| **Characteristic** | **Model 1** | |  | **Model 2** | |  | **Model 3** | |
| --- | --- | --- | --- | --- | --- | --- | --- | --- |
|  | **HR (95% CI)** | **P value** |  | **HR (95% CI)** | **P value** |  | **HR (95% CI)** | **P value** |
| **All-cause mortality** |  |  |  |  |  |  |  |  |
| ALI group | 0.37（0.28,0.48）） | <0.001 |  | 0.52（0.41,0.66）） | <0.001 |  | 0.55（0.43,0.69） | <0.001 |
| SII group | 1.76（1.31,2.38） | <0.001 |  | 1.76（1.34,2.31） | <0.001 |  | 1.80（1.37,2.36） | <0.001 |
| **Cardiovascular mortality** |  |  |  |  |  |  |  |  |
| ALI group | 0.40 (0.28, 0.57) | <0.001 |  | 0.65 (0.45, 0.94) | 0.0217 |  | 0.69 (0.47, 1.01) | 0.0581 |
| SII group | 1.93 (1.30, 2.88) | 0.0012 |  | 1.63 (1.08, 2.46 | 0.0193 |  | 1.68 (1.11, 2.55) | 0.0148 |
| **Competing risk of death** |  |  |  |  |  |  |  |  |
| ALI group | 0.36 (0.29, 0.45) | <0.001 |  | 0.51 (0.41, 0.65) | <0.001 |  | 0.54 (0.42, 0.68) | 0.0033 |
| SII group | 1.80 (1.38, 2.33) | <0.001 |  | 1.63 (1.25, 2.13) | <0.001 |  | 1.61 (1.23, 2.10) | <0.001 |

Cox proportional hazards models were employed to evaluate the relationships between ALL and SII with all-cause mortality rate. Multivariate Fine Gray competing risk regression models were employed to evaluate the relationships between ALL and SII with cardiovascular and non-cardiovascular mortality rate. Competing risk of mortality was defined as fatalities caused by factors other than cardiovascular disease. Model 1 was not altered. Model 2 was adjusted for age, gender, race, educational background, marital status, and the poverty-income ratio. Model 3 was adjusted for age, gender, race, educational background, marital status, poverty-income ratio, total cholesterol, high density lipoprotein, smoking status, alcohol consumption, cancer/malignancy, and coronary heart disease.

Abbreviations: ALI, advanced lung cancer inflammation index; SII, Systemic immune inflammation index; HR, hazard ratios; CI, confidence interval.


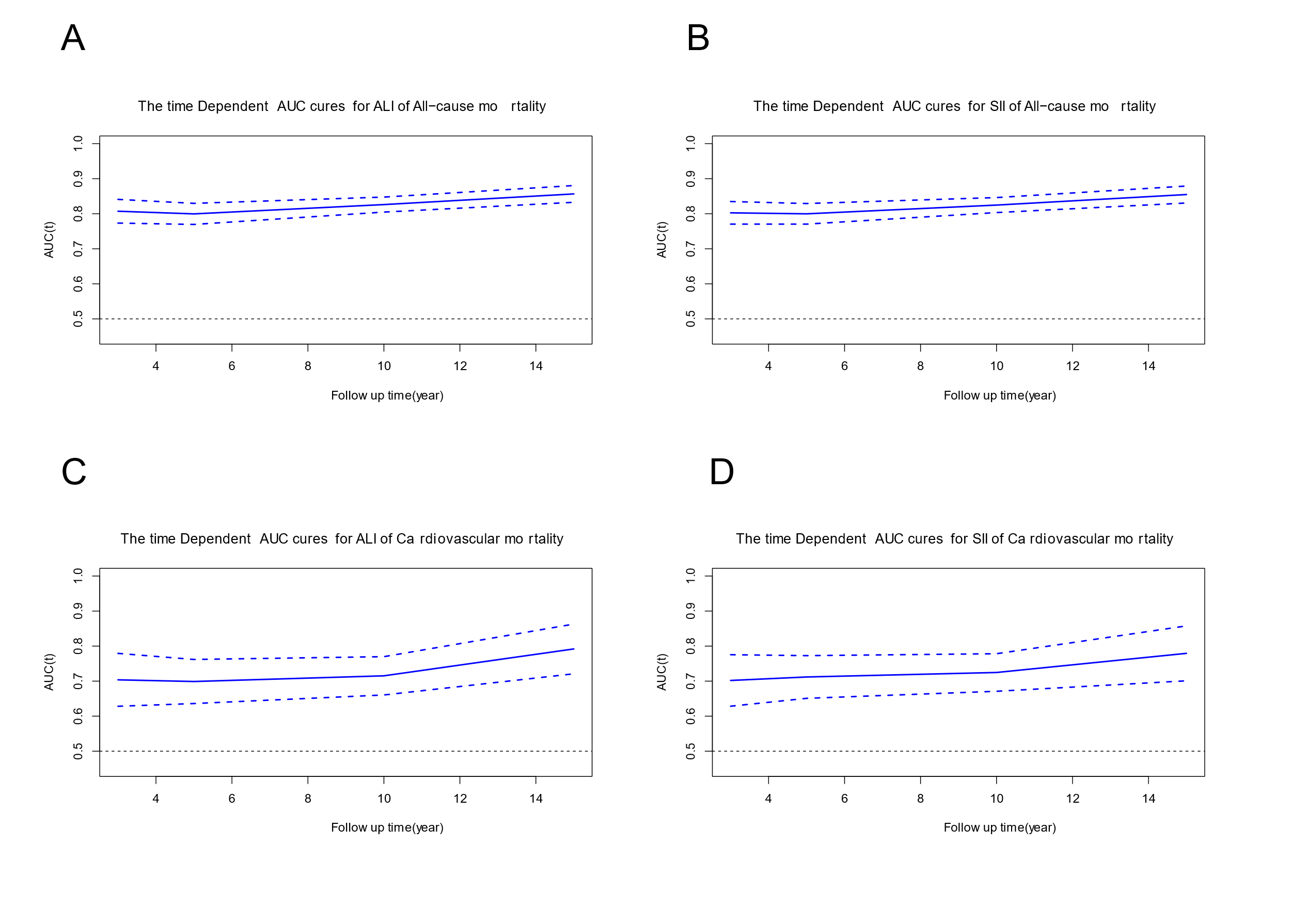


**Figure S1 The area under the ROC curve values (with a 95% confidence zone) for 3-,5-, 10-, and15- year survival predictions.**

A: ALI for all-cause mortality; B: SII for all-cause mortality; C: ALI for cardiovascular mortality; D: SII for cardiovascular mortality.

Abbreviations: AUC area under the curve, SII systemic immune-inflammation index, SIRI systemic inflammation response index
